# Neuroprotective Role of DING Protein in Normal Aging and Alzheimer’s Disease

**DOI:** 10.26502/aimr.0237

**Published:** 2026-03-17

**Authors:** Nune Darbinian, Armine Darbinyan, Paul Pozniak, Shohreh Amini, Michael E. Selzer

**Affiliations:** 1 Center for Neural Development and Repair, Department of Neural Sciences, Lewis Katz School of Medicine at Temple University, Philadelphia, PA 19140, USA.; 2 Department of Biology, College of Science and Technology, Temple University, Philadelphia, PA 19122, USA.

**Keywords:** Alzheimer’s Disease, Human DING, Eukaryotic DING, Phosphatase, Tau

## Abstract

**Introduction:**

Previously, we showed that the phosphatase DING extracted from St. John’s wort (p38SJ) is neuroprotective against EtOH-mediated toxicity in rat and human fetal neurons in vitro. Now, we assess the role of human DING in Alzheimer’s disease (AD). DING (p38SJ/p38hu), a member of the DING family of proteins, has been shown to be neuroprotective against cellular stress injury induced by alcohol, HIV-1, and in cancer cells. DING has demonstrated phosphatase activity on MAPK substrates, but its effect on Tau phosphorylation, which is involved in AD, has not yet been explored.

**Methods:**

Expression of DING protein levels was studied in human postmortem brain using histochemistry and quantitative western blot with ANOVA. Five patients with dementia, of whom 3 had AD neuropathology, a fourth had AD/Parkinson complex, and one had cerebrovascular dementia, were compared with 5 non-dementia controls.

**Results:**

DING was present in the neuronal cell bodies and processes of both normal and AD-affected human brain tissue. DING demonstrated phosphatase activity in PC12 cells (a cell line derived from rat pheochromocytoma) and inhibited Tau phosphorylation in these cells and in human brain tissue (both normal and AD). Increasing DING by transduction and overexpression in PC12 cells was associated with increased cell survival. In human brains (age=72–92 years), levels of endogenously expressed 38 kDa DING protein correlated positively with Tau dephosphorylation.

**Conclusions:**

Excess Tau phosphorylation leads to the formation of neurofibrillary tangles in neurons, a hallmark of neurodegeneration in Alzheimer’s disease. DING inhibits Tau phosphorylation and increases cell viability in non-proliferating neuronal cells overexpressing Tau, while reducing the viability of proliferating cells. Thus, DING may be neuroprotective in AD.

## 1. Introduction

Aging and Alzheimer’s Disease. Alzheimer’s disease (AD) is a leading cause of dementia in the elderly population of the US. The main pathologic findings in patients with AD are neurofibrillary tangles, neuronal inclusions composed of filamentous aggregates of hyperphosphorylated Tau, accumulation of plaques, and extracellular deposits of β-amyloid peptide [1,2]. These processes are associated with neuronal death, but the function of cellular systems aimed at preventing neuronal injury and preserving the integrity of neuronal cells in the aging brain remains largely unknown. There is an interesting link between AD and another neurodevelopmental disorder, fetal alcohol spectrum disorders (FASD). Interestingly, people with the most severe form of FASD, fetal alcohol syndrome (FAS), are estimated to live only 34 years (95% confidence interval, 31 to 37 years), and adults who are born with any form of FASD often develop premature aging. It was demonstrated that ethanol exposure alters Alzheimer's disease-related pathology, behavior, and metabolism in a mouse model of AD [3]. Alcohol use disorder was also linked to dementia [4]. On the molecular level, mitochondrial dysfunction and mitochondrial DNA (mtDNA) damage, hallmarks of aging, are also postulated to be central events in FASD, as ethanol (EtOH) can cause mtDNA damage, and consequently increase oxidative stress, and changes in the mtDNA repair protein 8-oxo guanine DNA glycosylase-1 (OGG1) [5]. Thus, many studies suggest that heavy alcohol consumption is linked to an increased risk of dementia in general and in AD in particular, as well as to acceleration of AD [6, 3]. However, there is little information on the prevention of EtOH-associated AD and other forms of dementia. Previously, we demonstrated that a plant protein, DING, exhibits phosphatase activity [7] and is neuroprotective against the toxic effects of EtOH in human brain and in fetal brain-derived exosomes (FB-E). Here, we investigate whether DING is present in both normal and AD human brain tissue, and if so, whether it affects the phosphorylation of Tau and has a neuroprotective effect associated with the abnormal hyperphosphorylation of Tau.

DING protein protects neuronal cells from injury. EtOH induces neuronal cell injury and death by dysregulating several signaling events that are controlled, in part, by activation of MAPK/ERK1/2 and/or inactivation of its corresponding phosphatase, PP1. We previously purified a novel 38 kDa protein containing a highly conserved N-terminus motif Asp-Ile-Asn-Gly (DING) that has phosphate binding activity (p38SJ/DING), from a callus culture of Hypericum perforatum (Saint John’s wort) [8, 9], and later human DING (p38hu) from human brain cells [10]. The DING family, named after the conserved N-terminal DING amino acid sequence, encompasses proteins that are implicated in the regulation of many functions in both eukaryotes and prokaryotes, e.g., genome expression and inflammatory responses to HIV [11, 12], and neuroprotection from EtOH-induced neuronal injury [7]. Homologous proteins have been found in all organism groups, both prokaryotic and eukaryotic [13], but it is still not known whether these proteins represent true variants or only minor mutations and cleavage products from only one highly conserved gene [14, 15, 16], with functions varying depending on the cell type and environment. The protein recovered from human brain (DINGhu), or other human phosphate binding protein, HPBP, is almost identical to that recovered from St. John’s wort and is recognized by the same antibodies [17, 18, 19, 20]. Treatment of primary cultures of rat neuronal cells with DING protected them against injury induced by exposure to EtOH in vitro and diminished the levels of pro-apoptotic proteins, including Bax and activated caspase-3 [7].

Similarly, DING protein was shown to protect neuronal cells from HIV-1-induced injury [6, 18] and mtDNA damage [5]. In addition, DING can inhibit the proliferation of cancer cells and glioblastoma multiforme [21]. To study whether DING can protect neuronal cells in Alzheimer’s Disease by its possible effects on the phosphorylation of Tau, we looked at the Tau status in the presence of DING in AD brain tissues, and in PC12 cells that are derived from rat adrenal pheochromocytoma and have been established as a model system for primary neuronal cells for their ability to respond to nerve growth factor (NGF). When treated with NGF, PC12 cells proliferation and develop processes like sympathetic neurons in primary culture while cell proliferation is inhibited [22, 23]. On the other hand, Tau is a key factor in neurofibrillary tangle formation, and using PC12 cells as a neuronal model [24, 25], we will determine if DING causes significant changes in Tau phosphorylation.

In the present study, we focused primarily on defining the changes in DING expression and its potential to exert protective effects in AD.

## 2. Materials and Methods

All procedures, tissues, and cell lines used in this study are summarized in Table 1.

### 2.1. Frozen brain tissue samples

of ten subjects (3 patients with AD [72–92 years], 1 patient with AD/PD [69 years], 1 patient with CD [83 years], and five controls [44–77 years]) were obtained from the National NeuroHIV Tissue Consortium (NNTC).

### 2.2. Rat pheochromocytoma PC12

cells were obtained from the American Type Culture Collection (ATCC, Manassas, VA). Cells were maintained in collagen-coated Petri dishes (100mm) in 10mL of Dulbecco’s Modified Eagle’s Medium (DMEM), accompanied by 15% fetal bovine serum and 2% horse serum with 10mg/mL gentamycin at 37 °C with 7% CO2 in a humidified incubation chamber. For NGF-treated cells, NGF was added two days after initial plating. Cells were cultured to 70% confluency. For serum-free cultures with transfection, cells were first plated using the described media, transfected as described below, and then the media was exchanged with serum-free DMEM after 8 hours of incubation at 37 °C with 7% CO2 in a humidified incubation chamber.

### 2.3. Plasmids.

YFP-p38SJ, CFP-Tau, GFP-Tau, and YFP-Tau were from Dr. Nune Darbinian and Dr. Judith Miclossy (Temple University). CFP-Tau was created by PCR amplification of a DNA fragment containing a coding region of Tau, cloned into pECFP-C1, pLEGFP-C1, or pEYFP-C1 plasmids (BD Biosciences Clontech, Mountain View, CA, USA). The nucleotides comprising all the plasmids were verified by DNA sequencing using an ABI automatic sequencer.

### 2.4. Transfection.

Transfection was performed according to the FuGENE 6 manufacturer’s protocol (Promega Corporation, Madison, WI). DNA plasmids were transfected at a 3:1 ratio of FuGENE 6 reagent: DNA for 100mm plates at a concentration of 3.33 μg per plate. The mixture of FuGENE 6 reagent and DNA was added directly to serum+ cell culture 1 day after plating. Transfection efficiency was assessed by fluorescence.

### 2.5. Treatment of PC12 cells.

PC12 cells were transfected with DING (3.3 ng/ml) and 24h post-transfection, were incubated with NGF for 48 h, for a total of 56 hours. The number of neurons and neuronal processes was measured in the presence or absence of serum or DING.

### 2.6. Preparation of protein extracts from PC12 cells and immunoblot analysis.

PC12 cells were incubated with NGF for 48 hours. For preparation of whole-cell extract from PC12 cultures, cells were removed from tissue culture plates with Trypsin, washed with cold phosphate-buffered saline (PBS), and solubilized in lysis buffer (50 mM Tris-HCl, pH 7.4, 150 mM NaCl, 0.1 % Nonidet P-40, and 1% protease inhibitors PI cocktail (Sigma). Cell debris was removed by centrifugation at 14,000 RPM for 5 min at 4^o^C, and the supernatant was collected in new Eppendorf tubes.

#### Western Blot Analysis.

After heating for 5 minutes at 95 °C, thirty micrograms of proteins were eluted with 6x SDS Laemmli sample buffer, separated by 10% sodium dodecyl sulfate-polyacrylamide gel electrophoresis (SDS-PAGE), and transferred to supported nitrocellulose membranes (Bio-Rad) for 2 hours at 4 °C at 55mA for 1 hour in 1x SDS buffer. The blots were blocked in 5% milk in PBS-T (PBS with 0.1% Tween 20) for 10 minutes and then incubated in primary antibody (1:1,000 in 5% milk in PBS-T) for 2 hours to overnight (incubations at 2 hours were performed at room temperature, but those exceeding 2 hours were performed at 4 °C). Blots were washed in PBS-T for 30 minutes (2x in fresh PBS-T for 15 minutes each) and then incubated in secondary antibody (1:10,000) in 5% milk in PBS-T for one hour. Blots were washed in PBS-T for 30 minutes (2x in fresh PBS-T for 15 minutes each) and then prepared for enhanced chemiluminescence detection using the ECL Plus system according to the manufacturer’s instructions (GE Healthcare, Piscataway, NJ) and exposed to X-ray film.

### 2.7. Quantitative Western Blot Assays. Preparation of total protein extracts from brain tissues and immunoblot analysis.

Frozen AD brain tissue samples of seven patients (3 patients with AD, 1 patient with AD/PD, 1 patient with CD, and two controls) were obtained from the National NeuroHIV Tissue Consortium (NNTC) and were homogenized in lysis buffer and continued with the same protocol as PC12 cell extracts. Homogenization, lysis, and western blotting were performed as previously described in our publications. The blots were subsequently washed three times, and the bound antibody was detected with the LI-COR system. Changes in protein levels in the human brain were measured by quantitative western blotting, as previously described [31]. The loading amount was determined by protein concentration. Proteins (30 μg) in Laemmli sample buffer were heated at 95 °C for 10 minutes, separated by gradient SDS-PAGE (4–20%), and transferred to an NC membrane. Proteins were detected using specific primary antibodies (1:1,000 dilution) and secondary IRDye^®^ dyes (IRDye^®^ 800CW Goat Anti-Rabbit and IRDye^®^ 680RD Goat Anti-Mouse Li-COR dyes, 1:10,000) with the Odyssey^®^ CLx Imaging System (LI-COR, Inc., Lincoln, NE, USA) using Odyssey software (LI-COR Biosciences, Lincoln, NE, USA). Band intensity (normalized to Grb2) was detected, visualized, and quantified using iS Image Studio^™^ Software version 3.1.

### 2.8. Antibodies.

Antibody specific for DING (rabbit polyclonal antibody) was obtained from Lampire Biological Laboratories, Inc. Pipersville, PA). Anti-α-tubulin clone B512 was obtained from Sigma-Aldrich (Sigma-Aldrich Co., St. Louis, MO). Antibodies specific for GFP (polyclonal against Aequorea Victoria recombinant full-length GFP or monoclonal BD “Living Colors”) and a loading control mouse monoclonal anti-Grb-2 antibody were obtained from BD Biosciences/Clontech (Palo Alto, CA, USA). Anti-Tau, which recognizes both non-phosphorylated and phosphorylated Tau, was from Santa Cruz Biotechnology, Inc. (Santa Cruz, CA). These primary antibodies were used in 1:1000 dilutions. Expression of the YFP-fusion protein, YFP-DING, was examined by Western blot analysis, using Living Colors full-length monoclonal antibody (BD Biosciences Clontech).

### 2.9. Phosphatase Assay.

The activities of YFP-DING were tested using the EnzoLyte pNPP protein phosphatase assay kit (colorimetric) upon incubation with pNPP for 1 hour at 37 °C, as per the manufacturer’s protocol (AnaSpec Inc., San Jose, CA). The kit uses pNPP, a colorimetric substrate for measuring the activity of tyrosine and serine/threonine protein phosphatases and ATPases, and spectrophotometry to determine the activity of phosphatases. As dephosphorylation occurs through the action of phosphatases, pNPP turns yellow, which can be detected by its absorbance at 405nm. Results are described in a graph showing absorbance at 405nm, which reflects phosphatase activity.

### 2.10. Cell viability assay (Trypan blue exclusion viability assay).

The trypan blue exclusion assay was performed to assess cell viability. This viability assay measures the percentage of a cell suspension that can exclude Trypan blue dye. In brief, the cell suspension was diluted 1:1 with 0.4% Trypan blue, and cells were counted with a hemocytometer. The averages and standard deviations are shown.

### 2.11. PC12 neuronal cell morphology analysis.

Neuronal cell complexity and outgrowth were evaluated after three days of transfection or treatment. The number of neurites in neuronal cells was determined by confocal microscopy, and the values were analyzed by ImageJ software (NIH). Experiments were repeated twice. The effects of DING overexpression on neurite outgrowth in neurons were compared with those of the control in 50 cells per group. The average neurite length is shown.

### 2.12. Immunohistochemistry.

Immunohistochemistry was performed using the avidin-biotin-peroxidase complex system according to the manufacturer's instructions (Vectastain Elite ABC Peroxidase Kit, Vector Laboratories, Inc., Burlingame, CA). Fluorescence detection was performed as described previously [8, 17]. Cells were seeded in poly-L-lysine-coated glass slide chambers. After 24 h incubation, cells were fixed and washed in PBS. Fluorescence images were captured using an inverted fluorescent Nikon microscope with deconvolution software (SlideBook 4.0.1.34; Intelligent Imaging, Denver, CO, USA). Fluorescence images of the cells were visualized using an inverted Olympus fluorescence microscope and IPLAB software. Contrast and brightness were adjusted equally for all images using Adobe Photoshop version 5.5.

### 2.13. Statistical Analysis.

Statistical analysis was performed using SPSS Statistics, version 25.0, released in 2017 for Windows by IBM Corp. (Armonk, NY, USA). All data are represented as the mean ± SD for all performed repetitions. Means were analyzed using a one-way ANOVA, with the Bonferroni correction applied where appropriate. Statistical significance was defined as p < 0.05. Sample numbers are indicated in the figure legends.

### 2.14. Ethics: Human Subjects.

Postmortem aged and AD brain tissues were received from the National NeuroHIV Tissue Consortium (NNTC). All investigators were trained annually to complete the Citi Program - Human Subject training, Biohazard Waste Safety Training, Bloodborne Pathogens Training, and all other required training.

Risk and Benefits. There were no risks associated with using frozen post-mortem brain tissues received from NNTC, as with any research study involving protected health information. Samples were de-identified before being sent to the lab for analysis.

There was no direct benefit to the research subjects from participation; however, there is potential benefit for future AD subjects and the general population. This research presents a valuable opportunity to advance the understanding, prevention, or alleviation of a significant issue affecting the health or welfare of AD patients.

## 3. Results

Previously, we demonstrated the presence of DING in human primary neuronal cultures and in fetal brain-derived exosomes [17]. We also revealed a neuroprotective function for exogenous DING against EtOH- and HIV-associated cell toxicity in vitro. In the present study, we used human aged brain tissues and brain tissues from AD patients (Table 1) to perform a series of molecular assays, immunocytochemistry, and immunohistochemistry to demonstrate the presence of DING in normal aged human brain tissues and in the PC12 cell line, and to study its neuroprotective features in AD brain tissues. A cell viability assay, also known as the Trypan blue exclusion viability assay, was performed in PC12 cells to measure the percentage of cells that can exclude the Trypan blue dye in proliferating cells (with serum in the media) or in differentiating cells (without serum). In differentiated cells, the neurite outgrowth was measured. Thus, in proliferating cells, we expect fewer viable cells in the presence of DING, whereas in non-differentiated cells, DING promotes neurite outgrowth.

### 3.1. DING is expressed in neurons of normal brains and in the brains of AD patients.

**Figure 1:**
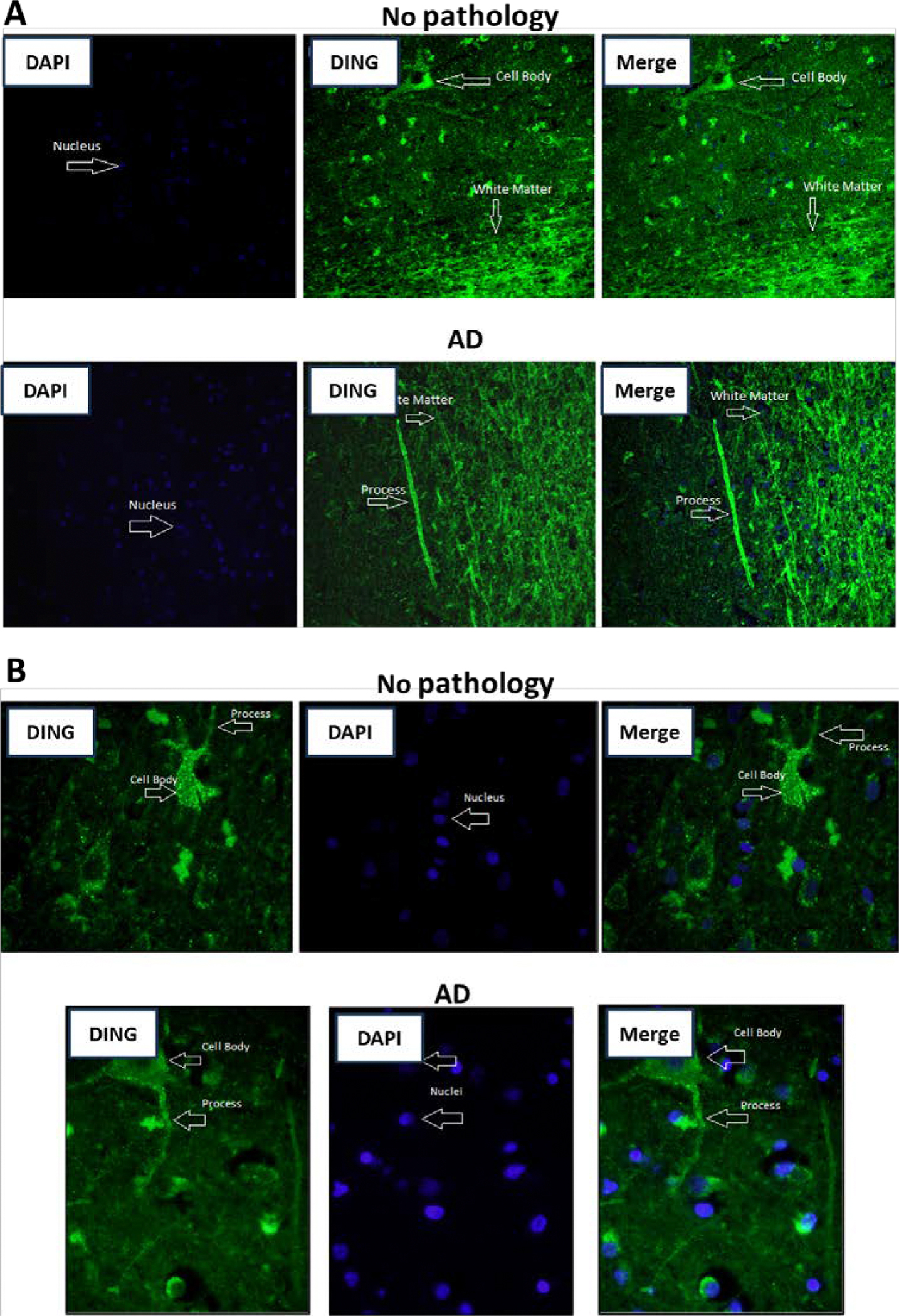
DING (green) is present in neurons of normal and AD brains. Evidence for the presence of DING in neurons in aging brain tissues. **A.** Immunohistochemistry staining of normal and Alzheimer’s disease human brain tissues. Green fluorescence represents DING, and blue is DAPI staining of DNA (nuclei); 20x magnification. **B.** DING protein (green) is expressed in neurons in aging and AD brain tissues; 40x magnification.

DING immunohistochemistry was performed on paraffin-embedded brain tissues from both normal and AD patients, both in the cortex and white matter (Figure 1A). In normal aging brain tissues, DING was localized mainly in the perinuclear space and in the perikaryal cytoplasm. However, in AD brains, DING was predominantly arrayed along neuronal processes (Figures 1A and 1B).

### 3.2. 38-kDa DING isoform is downregulated in AD brain tissues.

**Figure 2:**
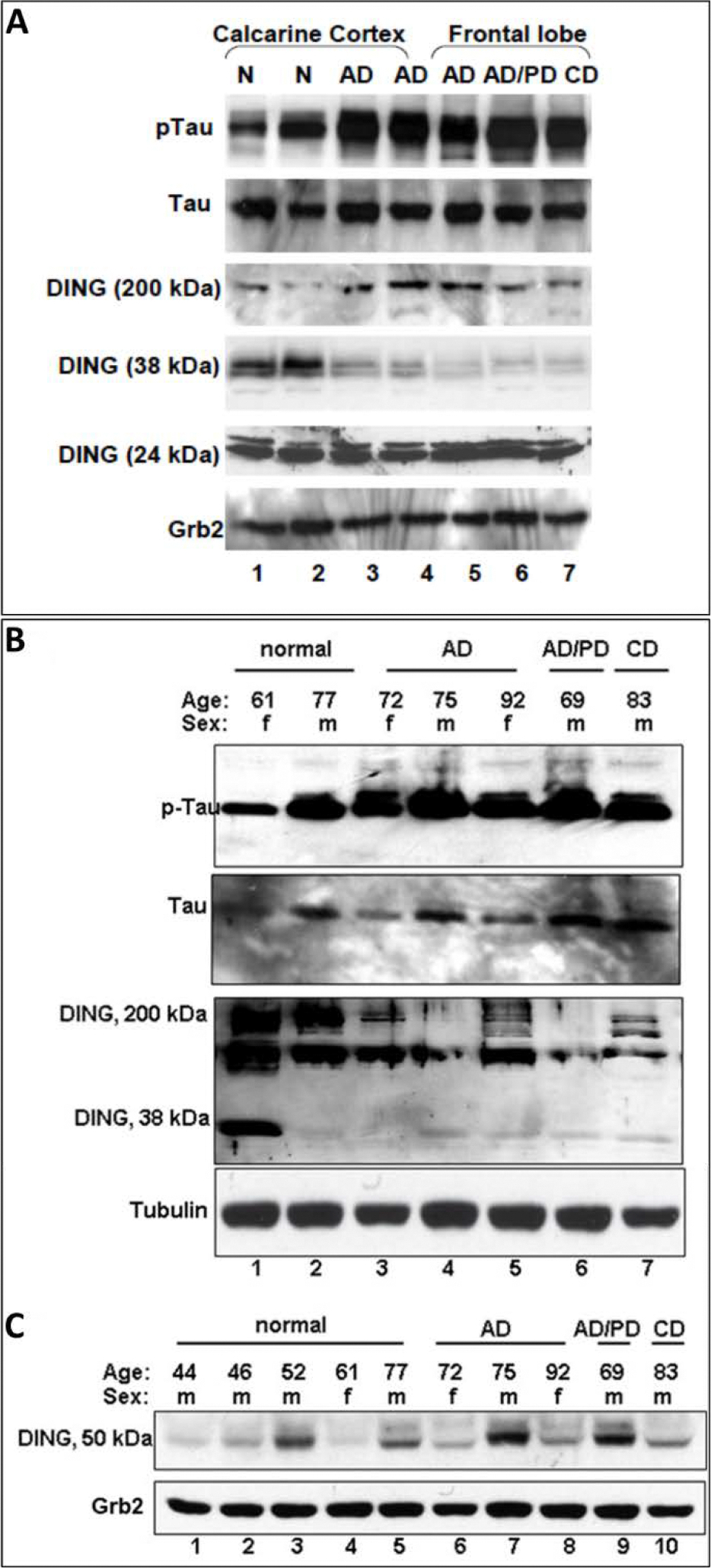
DING in normal and AD brain. Evidence for the presence of DING in aging brain tissues. The expression of DING in both normal and Alzheimer’s disease human brain tissues. AD, Alzheimer’s disease; AD/PD, Alzheimer’s/Parkinson’s complex; CD, cardiovascular disease; N, normal. Extracts were generated, and 40 μg of extract were loaded and separated on 10% SDS-PAGE. B. M, male; F, female. C. DING (50 kDa) isoform was expressed at a higher level in aged male cases. The National NeuroHIV Tissue Consortium (NNTC) provided frozen human brain tissue samples from healthy (n=5) and AD patients’ brains (n=4)/CD patient (n=1).

Western blot analysis was performed on homogenates of frontal lobe and calcarine cortex from normal brains and brains from patients with AD, AD/Parkinson complex, and cardiac disease. A relationship between DING levels and phosphorylated Tau in human normal and AD brain tissue is suggested by the Western blots of Figure 2. The expression of DING in AD human brain tissues was lower than in normal brain tissues or in brains from patients with cardiovascular disease (Figure 2A, panel 4, lanes 3–6). Interestingly, samples with low levels of 38-kDa DING had more phosphorylated Tau (Figure 2A, panel 1, lanes 3–6), while the level of total Tau (Figure 2A, panel 2, lanes 3–6) or housekeeping Grb2 (Figure 2A, panel 6, lanes 3–6) remained equal between all groups. Furthermore, phospho-Tau levels were higher in males than in females across all groups, including normal-aged brains (Figure 2B, panel 1). In addition, the 50-kDa DING isoform was expressed at higher levels in aged male cases (Figure 2C, panel 1). Phospho-Tau levels in brain tissue from a patient with dementia associated with cerebrovascular disease (CD) were slightly lower than in AD samples (Figure 2A, panel 1, lane 7). Phospho-Tau was also lower in male CD samples than in male AD samples (Figure 2B, panel 1, lane 7). Levels of the 50 kDa DING isoform were also lower in the CD sample, but the 38 kDa isoform of DING was not (Figure 2C, panel 1, lanes 10), suggesting the DING abnormality is specific to the pathology of AD, but to a lesser extent is also seen in other forms of dementia.

**Figure 3:**
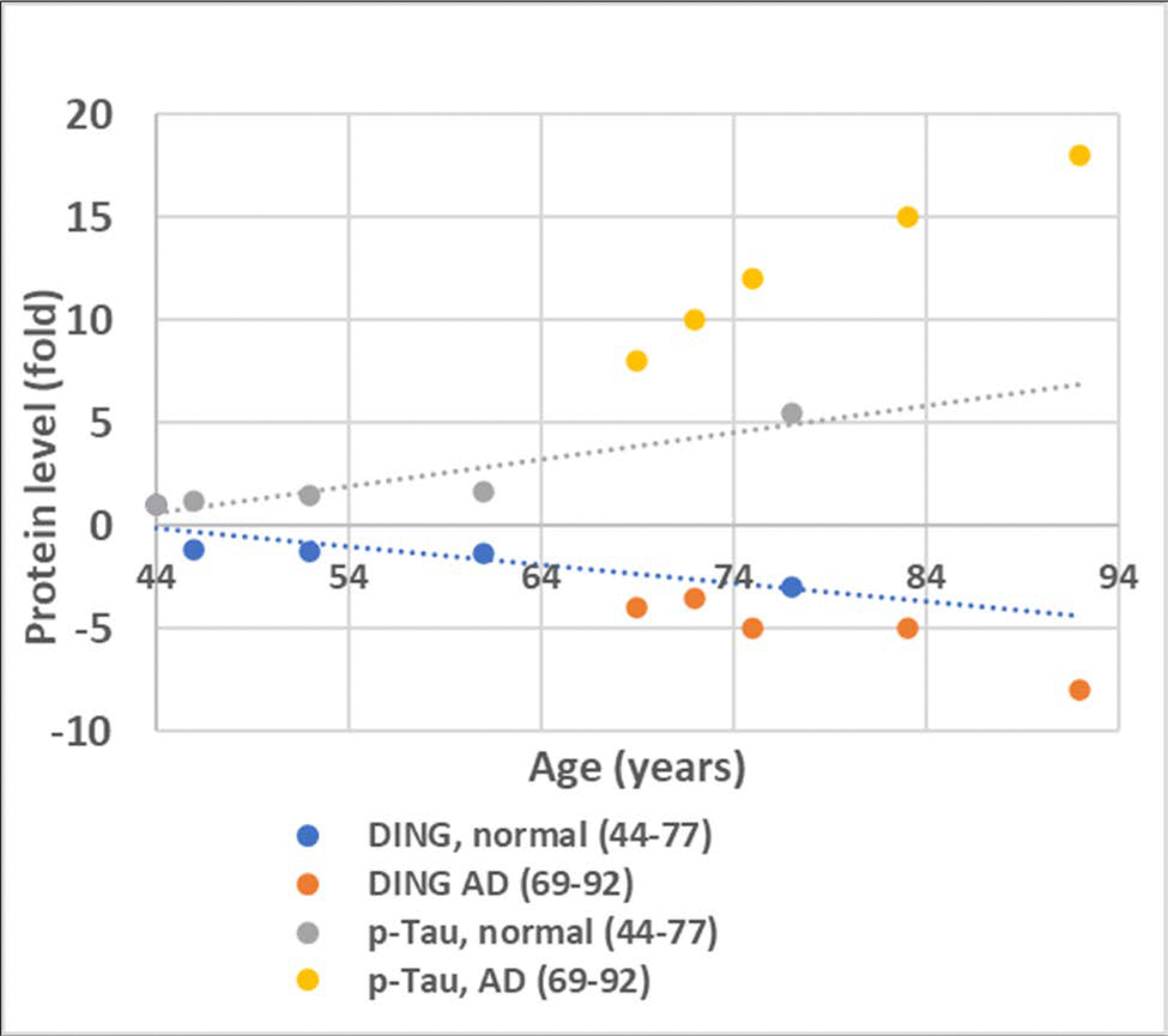
The relationship between DING expression and p-Tau levels in normal and AD brains.

Thus, while total Tau levels were similar in all groups, the levels of phosphorylated Tau were higher in four samples from AD patients, including patients with AD/Parkinson’s disease. The 200 kDa DING protein was also present in the four AD samples, including AD/Parkinson’s disease. Unlike phospho-Tau, 38kDa DING was plentiful in normal brain tissue, but was almost undetectable in AD. The 24 kDa DING protein was present in all samples. Thus, the loss of expression of the active form of DING, its 38-kDa isoform, correlated with the high levels of phosphorylated Tau seen in AD brains (Figure 3).

### 3.3. DING demonstrates phosphatase activity in PC12 cells, either proliferating (in the presence of serum) or non-proliferating (in the absence of serum).

**Figure 4:**
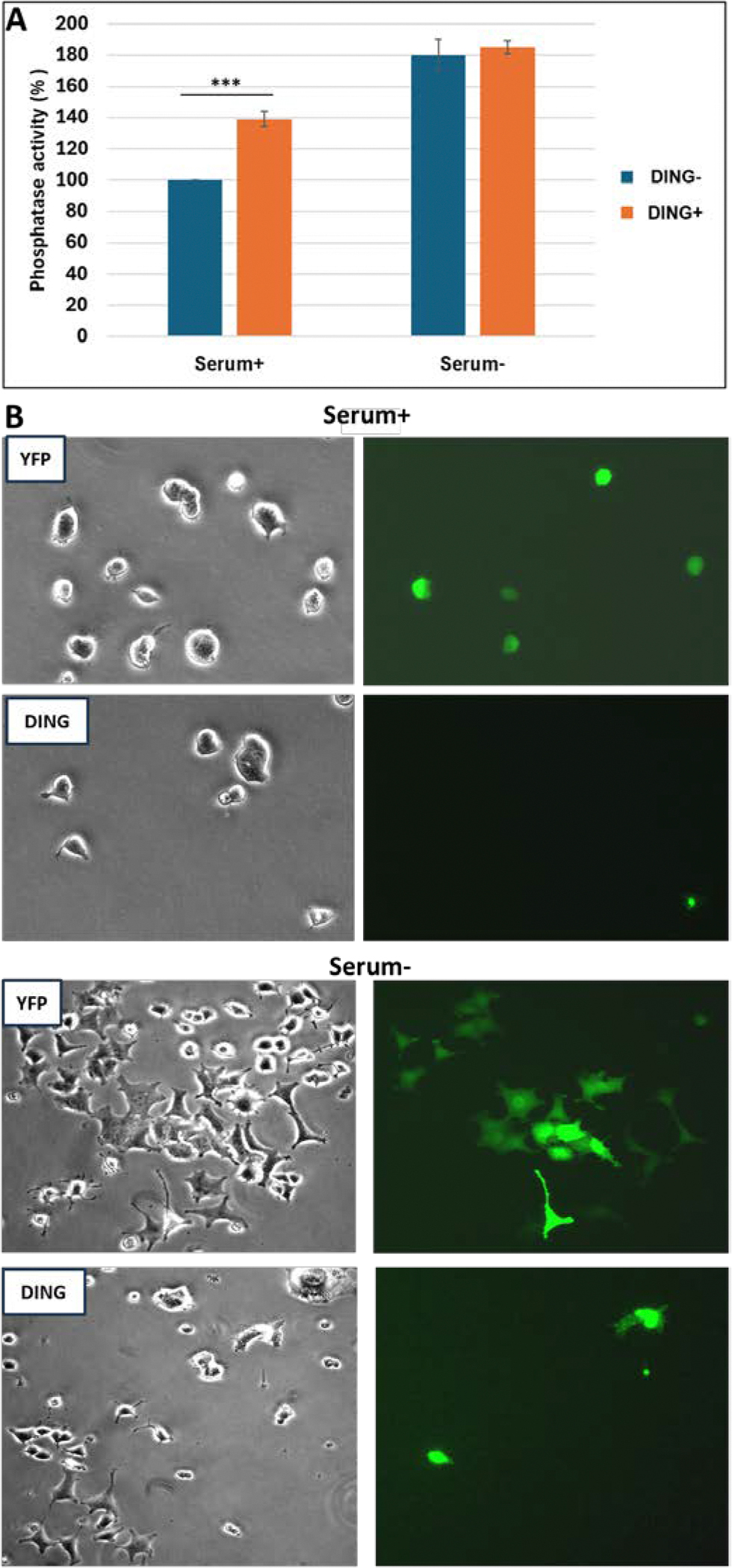
Phosphatase activity of DING in PC12 cells in the absence and presence of serum. Proliferating cells with growth factors (serum +), or non-proliferating cells (serum −). **A.** To examine whether DING, which contains a phosphate-binding domain, is involved in regulating cell proliferation or cell survival, we first investigated whether this protein exhibits phosphatase activity in neuronal cells. To that end, a phosphatase assay was performed using the Enzo Lyte pNPP protein phosphatase assay kit according to the manufacturer’s recommendation (Ana Spec Corporate, San Jose, CA). The activity of DING was tested by incubating the substrate with it at 37 °C for 1 hour. DING-associated phosphatase activity was higher in PC12 neuronal cells in the presence of serum (proliferating cells), while fewer changes were found in non-proliferating cells (serum−). Error bars represent the standard deviation from three independent readings. **B.** The effects of DING on cells. Cells were transfected with YFP- or YFP-DING-expressing plasmids in the presence or absence of serum. Fluorescent images of cells.

A phosphatase assay was performed on PC12 cells in the presence and absence of serum. In the presence of serum, the addition of DING increased phosphatase activity by 38%. Without serum, phosphatase activity of DING-free cells increased by 80% and with the addition of DING by 83% compared with controls consisting of cells in medium containing serum without DING (Figure 4). Proliferating cells with growth factors (serum +) or non-proliferating cells (serum −) were transfected with a DING-expressing plasmid. To examine whether DING, which contains a phosphate-binding domain, is involved in the dephosphorylation of proteins or the regulation of cell proliferation or cell survival, we investigated whether this protein exhibits phosphatase activity in neuronal cells. Phosphatase activity of DING was higher in PC12 neuronal cells in the presence of serum (in proliferating cells), while fewer changes were found in non-proliferating cells (serum −) (Figure 4A). In addition, there were fewer proliferating cells expressing DING, compared to the number of non-proliferating cells expressing DING (Figure 4B). Similar effects on the inhibition of proliferating cells have been demonstrated in primary glioblastoma cells [21].

### 3.4. DING, Tau, and serum affect cell viability synergistically.

**Figure 5:**
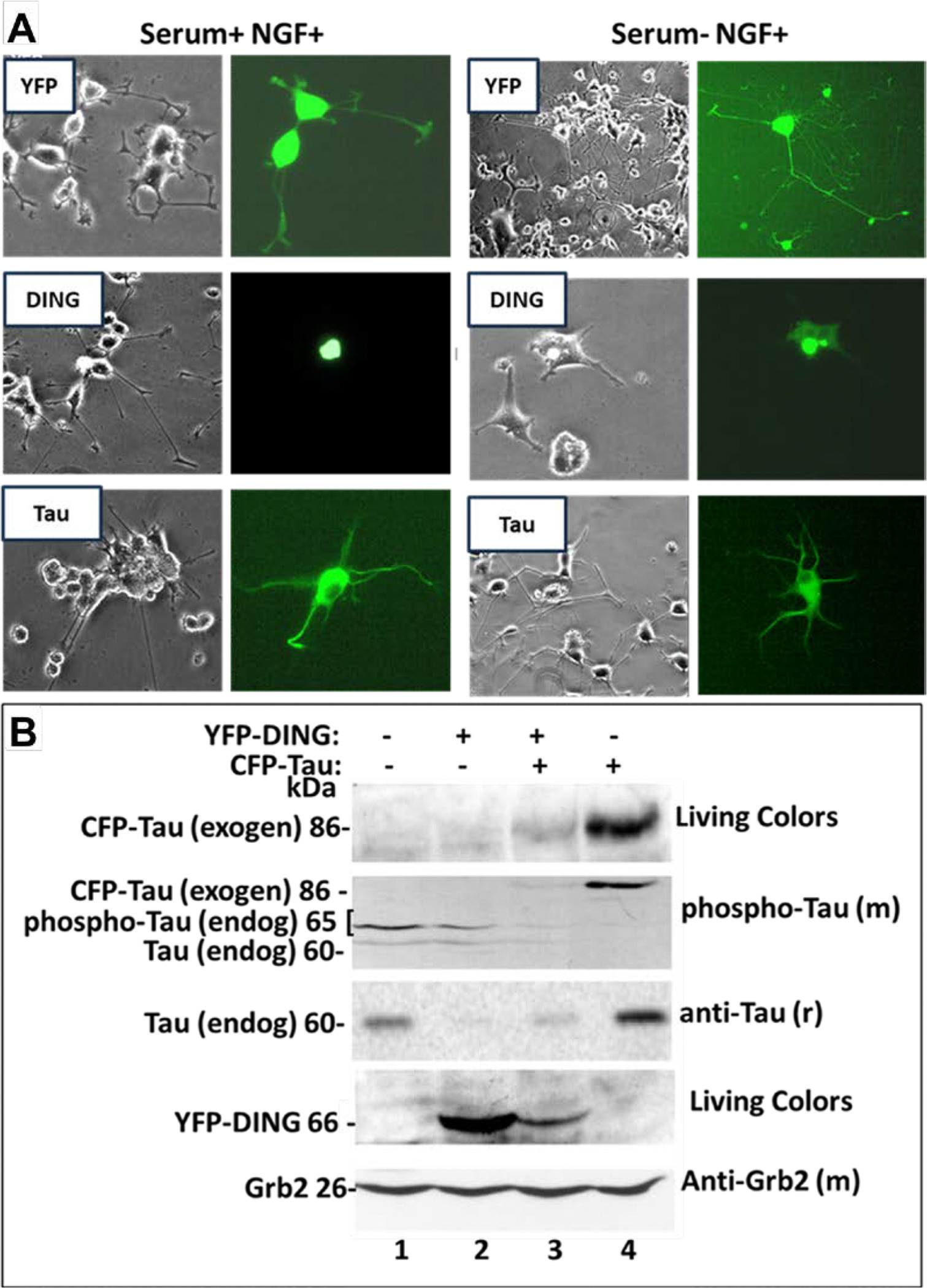
The effects of DING and Tau on PC12 cell proliferation. **A.** Effects of DING on neuronal cell morphology: phase and fluorescence images (magnification 200×) of neuronal cells incubated in the absence and presence of serum and/or NGF. Typical morphological illustration of neuronal cells for quantitative analysis of neuronal cell extension and complexity with NGF-treated cells. **B.** The effects of DING on Tau expression. Extracts were loaded at a concentration of 40 μg. Lane 1 does not have either YFP-DING or CFP-Tau; endogenous Tau is at 60 kDa, phospho-Tau is at 65 kDa; lane 2 only has YFP-DING (66 kDa); lane 3 has both YFP-DING (66 kDa) and CFP-Tau (86 kDa), and lane 4 only has CFP-Tau (86 kDa). Grb2 (24 kDa) was used as a control. The size of YFP or CFP is 26 kDa, the size of DING is 40 kDa, and the size of Tau is 60 kDa. The size of fusion proteins YFP-DING is 66 kDa, and CFP-Tau is 86 kDa.

PC12 cells proliferate in the presence of serum and differentiate in serum-free media [22]. Representative images of cells expressing DING in the presence or absence of serum (proliferation factor) or NGF (Nerve Growth Factor) demonstrate the suppression of cell proliferation by DING, as well as inhibition of neurite formation in serum-treated cells, while Tau did not have a visible effect on cell morphology and neurite formation (Figure 5A). DING and Tau also affected each other’s expression in PC12 cells (Figure 5B). Phospho-Tau level was low in the presence of DING (Figure 5B, lanes 2–3). At the same time, the transfected overexpressing DING level was also decreased in Tau-expressing cells (lane 3), suggesting an inhibitory effect of Tau either on DING expression or on the YFP promoter. To examine the effect of DING on the level of total Tau and phosphorylated Tau, whole-cell protein extracts were prepared from transfected PC12 cells, and Western blot analysis was performed. Figure 5B demonstrates the effects of DING on endogenous and exogenous Tau expression. Using the Living Colors antibody allowed the detection of exogenous Tau, as well as DING. The first panel shows no expression of Tau in lanes one and two, and in lane three, there is significantly less Tau than in lane four. In the second panel, there is less expression of Tau and less phosphorylation in the presence of DING. A change in exogenous and endogenous Tau was observed; endogenous Tau is expressed in lane 1, and a decrease in expression is shown in lane 2. Very little, if any, expression of endogenous Tau is observed in lane 3, and none is detected in lane 4. No exogenous Tau is observed in lanes one or two, very little in lane three, but it is expressed in lane four. In the third panel, total Tau expression is shown, with higher levels in lanes 1 and 4, but little to no expression in lanes 2 and 3. In the fourth row, DING is not expressed in lanes one or four, in the absence of DING, but is expressed in lanes two and three. The fifth row shows Grb2 expression in all conditions as an internal control.

**Figure 6:**
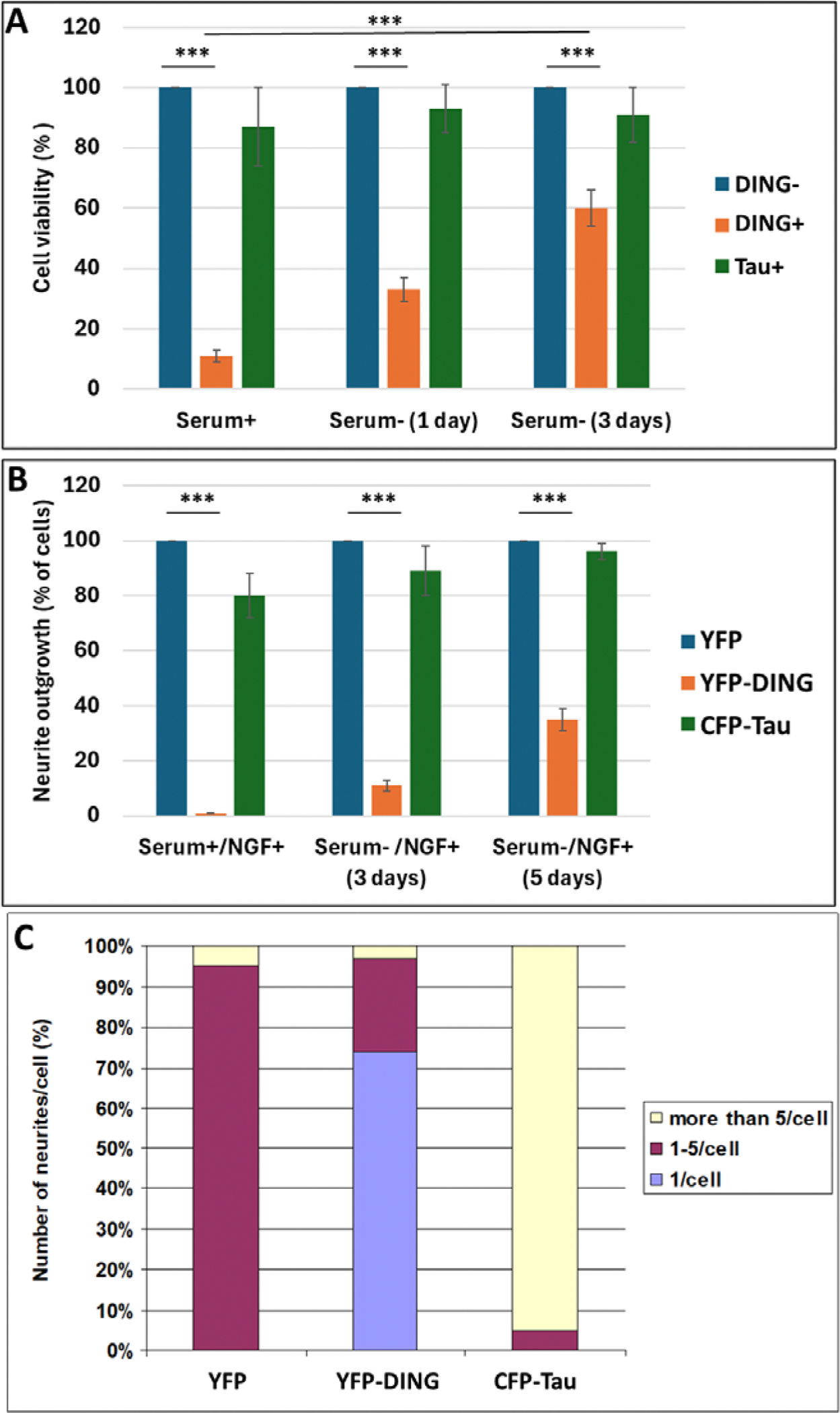
The effects of DING and Tau on the viability of PC12 cells. **A.** The effects of DING on cell viability in the presence or absence of serum in the media in PC12. YFP was used as a control in each condition and is referenced as 100% viability. In the presence of serum, cell viability is reduced. Cell survival was low, and the transfection efficiency of those live cells was also very low, as represented in the figure. Each was cultured in media containing serum for 2 days. Transfection was then performed, and serum-free media was exchanged after 3–8 hours of incubation. NGF was then added to all cells simultaneously. **B.** The effects of DING and serum on neurite outgrowth in PC12 cells when transfected with YFP, YFP-DING, or CFP-Tau. To determine the effects of each DING or Tau on neurite outgrowth, cells were transfected and induced to differentiate with NGF treatment. Neurites were only considered if they were longer than the cell body. YFP was used as a control in each condition and is referenced as 100% of cells containing neurites. **C.** The effects of YFP-DING and CFP-Tau on the number of neurites per cell. The average number of cells and neurites is shown in the graph. Bar 1 contains control YFP-expressing cells set at 10 cells.

Further, PC12 cells were incubated in serum-containing medium for 2 days, and then the serum was either withdrawn from the medium or left in the medium. The survival of DING-transfected cells (co-transfected with Tau) was then compared to that of control cells transfected with YFP. After one more day, in the presence of serum, viability was reduced in DING-expressing cells to 10% of that for PC12 cells that did not express DING (Figure 6A). The loss of viability was less (to only 30% of controls) when serum was removed for one day, and even less (to 60% of controls) after 3 days in serum-free medium. The addition of nerve growth factor (NGF) to induce the formation of neuronal processes had a similar effect as serum in DING’s anti-protective properties in slow-proliferative cells (Figure 6B).

Because phosphorylated Tau is a critical component of the neurofibrillary tangles found in AD, we looked for possible interactions between DING and Tau on neurite outgrowth (Figure 6). PC12 cells were transfected and induced to differentiate by treatment with NGF. Cells expressing YFP-DING showed no differentiation in media containing serum or NGF. Neurites were defined as processes longer than the diameter of the cell body. In the absence of serum or NGF, 10% and 30% of DING-expressing cells showed processes after 3 and 5 days of incubation, respectively. With YFP, 95% of cells formed 1–5 neurites per cell, and 5% had more than 5 neurites per cell. In the presence of DING, neurite outgrowth was reduced to 72% of cells having 1 neurite per cell, 26% of cells having 1–5 neurites per cell, and 2% of cells having more than 5 neurites per cell. Tau showed an increase in the number of neurites per cell at 96%, having more than 5 per cell, and 4% having 1–5 neurites per cell. DING could not promote the formation of processes in serum, unlike serum-free conditions (Figure 6B). Thus, for all cells, serum-free conditions showed more differentiation. YFP-transfected cells (controls) showed the most neurite outgrowth, with YFP-DING showing the least, although 1 neurite per cell was formed most in DING-expressing cells.

The effects of Tau, a key factor in neurofibrillary tangle formation, on neurite outgrowth in PC12 cells were similar to the effects of DING, but to a lesser extent. Whereas 96% of cells expressing YFP-DING plus CFP-Tau had 1–5 neurites/cell, and only 4% had more than 5 neurites/cell, most cells expressing DING alone (74%) had 1 neurite/cell, only 24% had 1–5 neurites/cell, and only 2% had more than 5 neurites/cell. Only 4% of cells expressing only Tau had 1–5 neurites/cell, while 96% had more than 5 neurites/cell, suggesting that Tau did not inhibit neurite formation (Figure 6C).

## 4. Discussion

To date, two major factors known to be involved in the neurodegeneration of Alzheimer’s disease are the formation of amyloid plaques and Tau neurofibrillary tangles [26]. Since amyloid deposits can be found in individuals with normal cognitive function, it has been speculated that dementia is more closely associated with neurofibrillary lesions, which develop within nerve cells of the cerebral cortex, hippocampal formation, and certain subcortical nuclei [27]. Neurofibrillary lesions in nerve cell bodies and apical dendrites take the form of neurofibrillary tangles (NFTs), consisting of paired helical filaments (PHFs) and straight filaments (SF). The microtubule-associated protein Tau constructs the filaments in its hyperphosphorylated state. Tau mRNA expression is found mainly in neurons, primarily in axons, although some have been described in oligodendrocytes [28]. The hyperphosphorylation of Tau in PHFs inhibits its binding to microtubules and is an early event in neurofibrillary lesion development. In vitro studies have shown the involvement of numerous protein phosphatases and kinases in abnormal Tau phosphorylation [25]. Tau is known to have phosphorylation sites for various kinases, including cyclin-dependent kinase 5 (Cdk5), extracellular signal-regulated kinases (ERKs), glycogen synthase kinase-3 (GSK-3), mitogen-activated protein kinases (MAPK), and c-Jun NH2-terminal kinases (JNKs) [29]. Elucidation of the mechanisms by which these kinases become involved may be useful for the development of therapeutic interventions in AD. Another avenue of AD research involving Tau phosphorylation is the activity of phosphatases involved in the dephosphorylation of Tau.

The involvement of DING proteins in a wide range of diseases enhances the potential therapeutic value of this specific protein family, further raising questions about the molecular mechanisms underlying these biological properties. Our observations reveal some important characteristics of DING and provide new insights into the potential regulation of disease-related DING proteins and their significance in treating human diseases.

To show a relationship between DING and Tau, and ultimately AD, DING must be present in AD tissues and must exhibit some effect on Tau phosphorylation, possibly by its phosphatase activity. The exact mechanism by which DING affects Tau phosphorylation remains unclear. However, the presence of DING expression certainly affects the phosphorylation of Tau. As shown in Figure 1, DING is present in both normal and AD human brain tissue, distributed throughout cell bodies and processes, and appears to be localized in the membranes of neuronal processes. The detectable presence of DING in AD tissue provides a reason for investigating its role in Tau phosphorylation. A limitation of this experiment is that the regions shown with DING localization have not been confirmed as damaged tissue. A co-staining of the tissues for phosphorylated Tau should be performed in future studies to establish regions damaged from AD progression. A negative correlation was found when DING expression was compared with Tau phosphorylation in normal human brain tissue and AD human brain tissue. As shown in Figures 2 and 3, the expression of DING is lower in AD samples when compared to normal tissue. Levels of the 50 kDa isoform of DING were lower in the CD sample (Figure 2). The opposite was observed for phosphorylated Tau. As expected, more phosphorylated Tau was expressed in AD than in normal brain tissues. More phosphorylated Tau was also was found in the CD sample than in normal brains, but less than in AD samples (Figure 2). Therefore, we investigated whether Tau phosphorylation is affected by DING. As Figure 4A shows, DING demonstrated phosphatase activity. Previously, DING was shown to be neuroprotective against metabolic stress and damage induced by alcohol. Therefore, we tested whether DING was neuroprotective in PC12 cells overexpressing Tau in medium with and without serum. PC12 cells, which are derived from a rat pheochromocytoma cell line, stop proliferating and die in the absence of serum. Cell viability in serum-free medium was increased in the presence of DING. Treatment with NGF prevents the death of PC12 cells in serum-free medium and promotes their differentiation into neuron-like cells. The difference in survival between control cells (transfected with YFP) and YFP-DING-transfected cells suggests that DING may have a pro-apoptotic effect in proliferative cancer cells. As the incubation time in serum-free conditions increased, the viability of Tau-transfected cells was also increased, suggesting that Tau may be involved in the late stages of cell signaling and survival. Neurite outgrowth also was affected in the presence of DING and Tau. In serum-free conditions, DING-transfected cells developed processes upon treatment with NGF. Most DING-expressing cells showed only one neurite per cell, suggesting that DING might be involved in axon development, although this has not yet been confirmed. Without serum, Tau overexpressing cells showed increases in the number of neurites per cell. There is a clear effect of serum on cell survival and neurite development in PC12 cells transfected with DING and Tau, and the interactions between these proteins require further investigation. Levels of both phosphorylated and total Tau were reduced in the presence of DING (p38SJ), suggesting that DING might be involved in both the expression of Tau and its phosphorylation, but the exact mechanisms for this have not yet been determined. A human neuronal cell model might be used to further determine the possible significance of DING in AD patients. Since DING is involved in regulating MCP-1 promoter activity [30], MCP-1 might be involved in AD pathogenesis. The current data may provide a possible model to more firmly establish a direct relationship between DING and AD.

## Conclusions

The data presented show that endogenous DING is expressed in normal human brains, but is dramatically reduced in the brains of patients with AD. While DING overexpression reduced the viability of proliferating PC12 cells, it increased the viability of non-proliferating neuronally differentiated cells. Moreover, in non-proliferating, differentiated PC12 cells overexpressing Tau, co-transfection with DING inhibited expression of Tau and also reduced its level of phosphorylation and enhanced cell viability. Since hyperphosphorylation of Tau leads to the development of neurofibrillary tangles, a pathological hallmark of AD, DING might have therapeutic potential in the treatment of AD.

## Figures and Tables

**Table 1:** Tissues and cell lines used in the study.

Tissues	Cell lines	Samples	Assays
**Human AD brain tissues. N=3; Ages (years): 72; 75; 92**	Rat PC12 cells	Protein lysates Tissue slides	Cell counting Phosphatase activity Cell viability assay Neurite outgrowth Western blot assay Immunohistochemistry Immunocytochemistry
**Human AD/PD brain tissue. N=1 Age (years): 69**			
**Human CD brain tissue. N=1 Age (years): 83**			
**Human normal brain tissues. N=5 Ages (years): 44; 46; 52; 61; 77**			

## Data Availability

This study collected laboratory data from post-mortem normal, aged people, and from people who were diagnosed with AD. We recognize that additional benefits from data sharing may arise in the future that are not apparent at this time. We are prepared to work specifically with NIH in addressing all requests for raw data. At present, we have not deposited the raw data in an existing databank but will make the data available to other investigators on request, in a manner consistent with NIH guidelines. Consistent with NIH policy, shared data will be rendered “free of identifiers that would permit linkages to individual research participants.” Intellectual property and data generated under this project will be administered in accordance with both University and NIH policies, including the NIH Data Sharing Policy and Implementation Guidance of March 5, 2003, and 0925–0001 and 0925–0002 (Rev 07/2022 through 01/31/2026). We will make deidentified data and associated documentation available to users only under a data-sharing agreement that provides (1) a commitment to using the data only for research purposes, (2) a commitment to securing the data using appropriate computer technology, and (3) a commitment to destroying or returning remaining samples after analyses are completed. The NIH implemented a new policy for Data Management and Sharing, effective on January 25, 2023 (https://grants.nih.gov/grants/guide/notice-files/NOT-OD-21-014.html). We will adopt that policy also. Data will also be available at https://www.mdpi.com/ethics accessed on January 1, 2026.

## Abbreviations:

AD: Alzheimer’s disease
ATCC: American Type Culture Collection
DING: p38SJ, p38hu protein; EtOH, ethanol
FAS: fetal alcohol syndrome
FASD: fetal alcohol spectrum disorders
NC: nitrocellulose membranes
NFTs: neurofibrillary tangles
NGF: nerve growth factor
NNTC: National NeuroHIV Tissue Consortium
PAGE: polyacrylamide gel electrophoresis
PC12: rat pheochromocytoma
PHFs: paired helical filaments
SF: straight filaments
